# Supplementary material for: Simultaneous Determination and Dietary Risk Assessment of 26 Pesticide Residues in Wheat Grain and Bran Using QuEChERS-UHPLC-MS/MS
Source: Foods. 2025 Dec 17;14(24):4351. doi: 10.3390/foods14244351 (PMC12732601; doi:10.3390/foods14244351)
Supplement: Supplementary file 1 [file foods-14-04351-s001.zip › foods-4021122-supplementary.pdf]

Table S1 Maximum residue limit (MRL), ADI ARfD, residue level and MR for wheat grain and bran for 26 pesticides

**Pesticides**	**ADI (mg kg^{-1} bw)**	**ARfD (mg kg^{-1} bw)**	**MRL (mg kg^{-1})**		**Residue level (mg kg^{-1})**		**MR (mg kg^{-1})**	
			**China**	**EU**	**Wheat grain**	**Wheat bran**	**Wheat grain**	**Wheat bran**
Acetamiprid	0.07	0.1	0.5	0.1	＜0.005	＜0.01-0.0221	＜0.005	＜0.01
Azoxystrobin	0.2	-	0.5	0.5	＜0.005-0.3925	＜0.01-0.6188	0.011175	1.098
Bensulfuron-methyl	0.2	-	0.02	0.01*	＜0.005-0.0405	＜0.01-0.2259	＜0.005	＜0.01
Chlorotoluron	0.04	-	0.1	0.1	＜0.005-0.0084	＜0.01-0.4057	＜0.005	＜0.01
Clothianidin	0.1	0.6	0.02	0.02*	＜0.005-0.0177	＜0.01-0.3212	＜0.005	0.0327
Cyproconazole	0.02	0.06	0.2	0.1	＜0.005	＜0.01	＜0.005	＜0.01
Diazinon	0.005	0.03	0.1	0.01*	＜0.005	＜0.01-0.4116	＜0.005	＜0.01
Dichlorvos	0.004	0.1	0.1	0.01*	＜0.005	＜0.01-0.5006	＜0.005	＜0.01
Difenoconazole	0.01	0.3	0.1	0.3	＜0.005-0.0216	＜0.01-0.2448	＜0.005	1.001
Diflufenican	0.2	-	0.05	0.02	＜0.005	＜0.01-0.8012	＜0.005	＜0.01
Dimethoate	0.002	0.02	0.05	0.01*	＜0.005	＜0.01-0.2457	＜0.005	＜0.01
Diniconazole	0.005	-	0.2	0.01*	＜0.005	＜0.01-0.2330	＜0.005	＜0.01
Epoxiconazole	0.02	-	0.05	0.01*	＜0.005-1.7851	＜0.01-0.3889	＜0.005	1.089
Fenaminstrobin	0.069	-	0.1*	-	＜0.005	＜0.01-0.5968	＜0.005	＜0.01
Flumetsulam	1	-	0.05	-	＜0.005	＜0.01-0.4643	＜0.005	＜0.01
Hexaconazole	0.005	-	0.1	0.01*	＜0.005-0.0083	＜0.01-0.2709	＜0.005	1.002
Imidacloprid	0.06	0.4	0.05	0.01*	＜0.005-0.0096	＜0.01-0.2488	＜0.005	0.02095
Isoproturon	0.015	-	0.05	0.01*	＜0.005-0.0096	＜0.01-0.3004	＜0.005	＜0.01
Kresoxim-methyl	0.4	-	0.05	0.08	＜0.005-0.5450	＜0.01-0.3762	＜0.005	1035
Metsulfuron-methyl	0.25	-	0.01	0.01*	＜0.005-0.0164	＜0.01-0.3428	＜0.005	＜0.01
Myclobutanil	0.03	0.3	0.1	0.01*	＜0.005-0.068	＜0.01	＜0.005	＜0.01
Pirimicarb	0.02	0.1	0.05	0.05	＜0.005	＜0.01	＜0.005	＜0.01
Tebuconazole	0.03	0.3	0.05	0.3	＜0.005-0.2924	＜0.01-2.1883	＜0.005	0.2037
Thiamethoxam	0.08	1	0.1	0.05	＜0.005	＜0.01-0.1328	＜0.005	＜0.01
Triazophos	0.001	0.001	0.05	0.02*	＜0.005	＜0.01	＜0.005	＜0.01
Trichlorfon	0.002	-	0.1	0.01*	＜0.005	＜0.01	＜0.005	＜0.01

*: Temporary limit.
